# The New Preparations of Rubber

**Published:** 1868-03

**Authors:** 


*The New Preparations of Rubber.*—In the February Number of this Journal we called the attention of our readers to two new kinds of dental rubber, the vulcanizing or hardening of which does not interfere with the Goodyear and Cummings' patents.

Since then we have received from Snowden & Cowman of this city, who are the agents for the sale of both preparations, samples of each, and the following is the result of our experiments with them.

*Simpson's Rubber*, from the Porter Manufacturing Company. Our readers will remember that Hale's rubber, under the Simpson patent contains only two and a half ounces of sulphur to a pound of rubber; the minimum fixed by the Goodyear patent being four ounces. The process of preparing the model, flask, &c., is the same with the Simpson rubber as with the Goodyear, except that a somewhat longer time is required for vulcanizing it. In the first experiments made with it, we ran the mercury up to 340° and kept up this degree of steam for fifty five minutes. The result was very satisfactory, and as far as we can judge from appearance and a trial of its strength, it is in every respect equal to the American Company's Rubber Other experiments were made by subjecting this rubber for a longer time to a lower degree of steam; heating up gradually 30 minutes to 320° and keeping it at this degree for three hours and a half which gives a lighter color and rather more elasticity. The directions accompanying this rubber call for 320° for three hours and thirty minutes.

*The Iodized Rubber.*—This rubber as we informed our readers in the February Number of Journal contains no sulphur, but is hardened by "bromide of iodine," the process being patented by Newbrough and Fagan. It is made by adding to iodine one-half its weight of bromine, the result being the proto-bromide of iodine, which when combined with rubber in the proportion of three ounces of the paste to a pound of the gum, produces a composition which will harden on being subjected to a dry heat of 310° Fah. for one hour.

The following comparison with Dental Vulcanite is given.

| Iodized Rubber before baking. | | | Vulcanite before Vulcanizing. | | |
|---|---|---|---|---|---|
| Rubber | Oz. | 16 | Rubber | Oz. | 16 |
| Bromide of Iodine | " | 3 | Sulphur | " | 8 |
| Oil of Vitriol | " | 1 | Vermilion | " | 20 |
| Vermilion | " | 6 | Total, | | 44 |
| Total, | | 26 | | | |

| After baking. | | | After Vulcanizing. | | |
|---|---|---|---|---|---|
| Rubber | Oz. | 16 | Rubber | Oz. | 16 |
| Vermilion | " | 5¾ | Sulphur | " | 9¼ |
| Sulphur of Iodine | " | ¼ | Vermilion | | 18¾ |
| Total, | | 22 | Total, | | 44 |
| Resume: Rubber 16, Foreign matter 6. | | | Resume: Rubber 16, Foreign matter 28. | | |

The following rules for baking the Iodized Rubber must be followed: After the wax is removed, the open flask is laid on the stove and *thoroughly dried* of all moisture. The case is then packed at a *low dry heat*, when it is put in the oven and the light applied. The heat is allowed to rise to 310° Fah. within 15 or 20 minutes, and held at this degree for one hour, after which time it is is increased to 380° F., for one hour longer. Ten minutes after the light is put out the flask may be removed from the oven and cooled with cold water.

For repairing, the piece is encased in the usual manner, but the "top half" of the flask only is dried.

The collodion varnish is used on the surface of the plaster as with the other preparations of rubber, and it is recommended to add one-fifth of pumice stone or fine sand to the plaster, and salt or a drop or two of sulphuric acid to the water used for mixing the plaster.

The iodized rubber will not harden on a moist plaster surface, hence the necessity to thoroughly dry the plaster in the flask. Our first experiment with the iodized rubber was made with the common vulcanizer and was a failure, it being necessary to use the oven prepared expressly for this compound. This oven consists of a cast-iron, cone-shaped boiler at least two inches thick in the bottom with a heavy lid placed over it; no packing or screws being necessary, as dry heat is used. The weight of the lid resting on the top of the boiler or oven is sufficient to confine the heat, which is generated by a small lamp placed under the apparatus.

Our experiments with the apparatus just described were perfectly successful, and the result was a hardened base of a somewhat darker color than the dental vulcanite, capable of being highly polished and apparently, as we have not as yet had an opportunity of judging by a trial in the mouth, as strong and durable as the best dental vulcanite base.

